# Sentinel Lymph Node Biopsy Mapped With Carbon Nanoparticle Suspensions in Patients With Breast Cancer: A Systematic Review and Meta-Analysis

**DOI:** 10.3389/fonc.2022.818812

**Published:** 2022-03-28

**Authors:** Yan Jiang, Jiayang Li, Baolin Chen, Yuxiang Bao, Chengmin Luo, Yi Luo, Taolang Li, Junyuan Lv, Xiaoming Cheng

**Affiliations:** ^1^Breast and Thyroid Surgery, Department of General Surgery, The Affiliated Hospital of Zunyi Medical University, Zunyi, China; ^2^Drug Clinical Trial Institution, The Affiliated Hospital of Zunyi Medical University, Zunyi, China

**Keywords:** meta-analysis, diagnosis, sentinel lymph node biopsy, carbon nanoparticle suspensions, breast cancer

## Abstract

**Background:**

The mapping method represents a crucial factor affecting the rate of sentinel lymph node detection in breast cancer. We carried out this meta-analysis to assess the clinical utility of carbon nanoparticle suspensions (CNSs) in guiding sentinel lymph node biopsy (SLNB) for breast cancer patients.

**Methods:**

Electronic databases, which comprised the China National Knowledge Infrastructure, the Wanfang electronic database, the Cochrane Library, EMBASE, and PubMed, were explored to identify relevant studies from database inception to July 2021 that studied the detection rate of CNSs-guided SLNB. A meta-analysis was performed to generate pooled sensitivity, specificity, positive likelihood ratio (PLR), negative likelihood ratio (NLR), a summary receiver operator characteristic curve (SROC), and a diagnostic odds ratio (DOR).

**Results:**

A total of 33 publications that enrolled 2,171 patients were analyzed. The pooled sensitivity, specificity, PLR, and NLR were 0.93 (95% CI: 0.91–0.95, *I^2^* = 0.0%), 0.99 (95% CI: 0.98–0.99, *I^2^* = 56.5%), 42.85 (95% CI: 29.73–61.77, *I^2^* = 47.0%), and 0.09 (95% CI: 0.07–0.11, *I^2^* = 0.0%), respectively. The area under the curve (AUC) of the SROC curve was 0.98. There were no significant differences when analyzed based on the dose and site of CNS injection. There was significant publication bias among the included publications based on Deeks’ funnel plot [Slope (Bias) = −7.35, *P* = 0.00]. Nonetheless, the sensitivity analysis identified the results to be reliable and stable.

**Conclusion:**

This meta-analysis highlights the accuracy and feasibility of using CNSs for SLNB in patients with breast cancer. Clinically, the identification and predictive values of CNSs as an optimal tracer for SLNB remains undisputed.

## Introduction

The modern era of breast cancer surgery is progressing towards the direction of minimally invasive treatment. Previously, axillary lymph node dissection (ALND) represented an indispensable treatment component for breast cancer. However, the current standard of care for axillary staging is SLNB. The sentinel lymph node refers to the first axillary lymph node draining the tumor site and may potentially harbor metastatic deposits (1). SLNB is mainly determined by evaluating the SLN status to determine whether ALND is required. SLNB allows for careful selection of patients who are candidates for ALND. SLNB is as effective as ALNB but has the benefits of lower postoperative complications such as arm lymphedema and sensory loss (2–5). The mapping method is a crucial factor that determines the positive and negative detection rates of SLNB in breast cancer. SLNB techniques incorporate the use of either blue dye (BD) or radioisotopes (RI) (6). The RI method requires specialized equipment, authorized radiation protection areas, and nuclear medicine licensing, thus limiting the widespread use of this approach. BD, on the other hand, is a cost-effective method for SLNB but possesses a lower detection rate (7).

The past decade has seen a surge in research in the field of nanomaterials and nanotechnology. Several novel diagnostic and therapeutic techniques in the field of medicine have begun to incorporate nanobiotechnology. CNSs is a 150 nm nanoparticle lymphatic tracer made up of polymeric carbon granules and has been approved for clinical usage by the Chinese Food and Drug Administration (CFDA). CNSs selectively populate the lymphatic system (diameter: 120–500 nm) over the vascular system (diameter: 20–50 nm), given its permeability and molecular size (8). CNSs have received substantial attention over the recent years, especially with regards to their postulated benefits in lymphatic mapping. Thus, the aim of our analysis was to assess the effectiveness of CNSs for SLN mapping in breast cancer.

## Materials and Methods

### Literature Search

A systematic literature search was carried out on the China National Knowledge Infrastructure, the Wanfang electronic database, the Cochrane Library, EMBASE, and PubMed to extract all related papers present from database inception until July 2021. The medical subject heading (MESH) terms used were as follows: breast neoplasm, breast carcinoma, breast tumor, breast cancer, carbon nanoparticle, nano-carbon, carbon nanoparticles suspensions, CNSs, sentinel lymph node biopsy, and SLNB.

### Inclusion and Exclusion Criteria

The inclusion criteria were as follows:

1. Patients with breast cancer who had clinically negative lymph nodes.
2. The concurrent use of CNSs and other modalities for SLNB mapping.
3. The availability of diagnostic method and clinicopathological data.
4. The SLNB as the main study topic.
5. The reported primary data were sufficient to calculate totals of true negative (TN), false negative (FN), false positive (FP), and true positive (TP).

The exclusion criteria were as follows:

1. Letters, editorials, review articles, and case reports.
2. Overlapping information between studies.

### Data Extraction and Quality Assessment

All studies were reviewed by two independent reviewers in order to extract the relevant data. A third reviewer was consulted to reach a consensus in case of a disagreement. A datasheet containing the following information was compiled: year of publication, author, age, dose of CNSs, injection site, TN, FN, FP, and TP values. The Quality Assessment of Diagnostic Accuracy Studies (QUADAS-2) protocols were referenced for quality assessment of the selected studies (9). These guidelines evaluate the degree of biases in the included studies across four major domains that included flow and timing, reference standard, index test, and patient selection. The highest possible score is 14, which indicates high study quality.

### Statistical Analysis

The STATA version 15.1 (Stata Corporation, College Station, Texas, USA) and Meta-Disc version 1.4 Software (XI Cochrane Colloquium; Barcelona, Spain) was utilized for this meta-analysis. The degree of heterogeneity among the studies was estimated using *I^2^*, while heterogeneity itself was assessed with the Chi-square-based Q statistic test. Heterogeneity was interpreted as being statistically significant when *I^2^* >50% or *P <*0.05. The fixed-effect model (Mantel–Haenszel) was used in cases of no study heterogeneity. In cases where there was study heterogeneity, a random-effect model (DerSimonian and Laird) was implemented.

Study sensitivity, specificity, PLR, NLR, and DOR were evaluated using a bivariate meta-analysis model. A suitable statistical analysis model was first used to calculate the estimates with the corresponding 95% CI. The AUC and SROC of these models were also determined. A higher diagnostic effect was recognized in results that had an AUC closer to 1.0. Publication bias was determined with the Deek test for funnel plot asymmetry.

## Results

### Characteristics of Identified Studies

**Figure 1:**
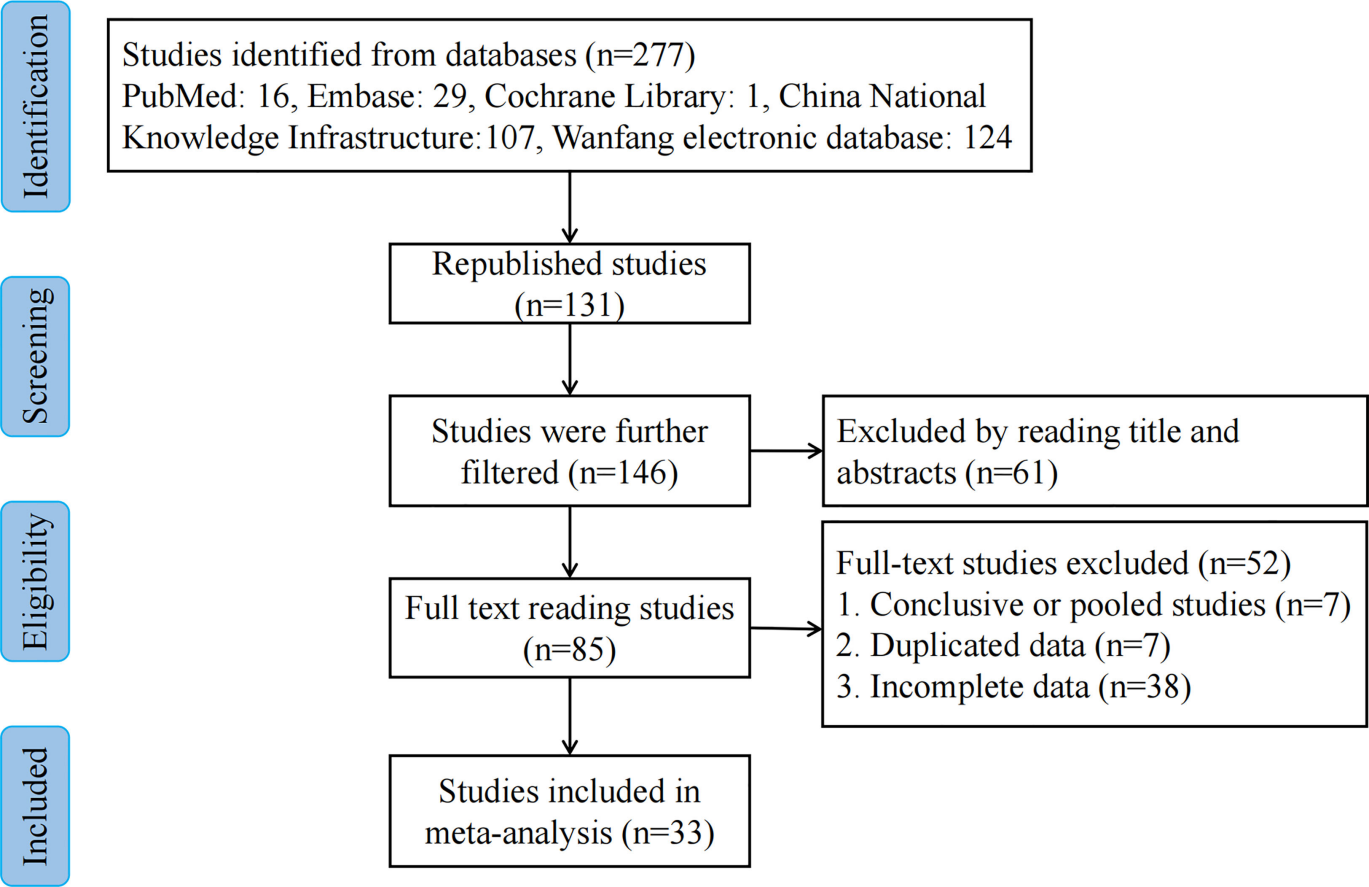
Flowchart of the study selection process.

We extracted 277 potentially relevant publications. Of these, 131 duplicates were removed, and 61 were deemed irrelevant based on screening titles and abstracts. A total of 85 remaining full-text articles were then scrutinized for eligibility (**Figure 1**). Another 52 articles were additionally excluded: 7 articles were excluded due to duplicate use of the same data, 7 articles were summary and summary data, while 38 articles contained incomplete data. Finally, 33 studies (10–42) including 2,171 patients were included in our meta-analysis. The amount of CNSs injected ranged from 0.2 to 2 ml. Peritumoral CNSs injection for SLNB was used in 3 studies, subareolar CNSs injection was used in 15 studies, and both peritumoral and subareolar CNSs injection were used in 14 studies. **Table 1** depicts the characteristics of the identified papers.

**Table 1:** Detailed characteristics and QUADAS scores of the included study.

Study	Year	No. of patients	Age (years)	Dose of CNSs (ml)	Injection site	TP	FP	FN	TN	QUADAS
Liu et al. (10)	2019	59	51.5 ± 9.7	1 ml	Peritumoral	15	1	2	41	12
Wu et al. (11)	2019	46	53.63 ± 8.57	2 ml	Mixed	15	0	1	30	12
Xia et al. (12)	2019	86	51.37 ± 5.23	1 ml	Mixed	25	0	1	60	12
Gao et al. (13)	2018	58	47.2 ± 15.1	2 ml	Mixed	24	0	2	32	12
Li et al. (14)	2018	47	43.25 ± 10.15	2 ml	Subareolar	15	0	1	31	12
Wang et al. (15)	2018	77	NA	0.6 ml	Mixed	20	0	1	56	12
Qi et al. (16)	2018	52	50.2 ± 9.5	1 ml	Mixed	16	0	1	35	12
Zhang et al. (17)	2018	91	NA	1 ml	Subareolar	47	0	2	42	12
Yang et al. (18)	2018	136	50.9 ± 10.8	1 ml	Mixed	55	0	4	77	12
Zou et al. (19)	2017	86	NA	0.5 ml	Mixed	23	0	2	60	12
Wang et al. (20)	2017	77	NA	0.5 ml	Subareolar	28	0	1	48	12
Wang et al. (21)	2017	53	NA	0.6 ml	Mixed	12	0	1	40	12
Yue et al. (22)	2017	50	NA	0.4 ml	Subareolar	22	0	2	26	12
Zhang et al. (23)	2017	140	NA	NA	Mixed	20	0	2	118	12
Kong et al. (24)	2016	56	57.2 ± 11.1	2 ml	Subareolar	15	2	1	38	11
Sang et al. (25)	2016	42	NA	1 ml	NA	17	0	3	22	12
Kong et al. (26)	2016	63	NA	0.2–0.5 ml	Mixed	13	8	1	41	12
Huang et al. (27)	2016	83	NA	1 ml	Subareolar	16	1	2	64	12
Liu et al. (28)	2016	83	NA	NA	Subareolar	24	0	3	56	12
Chen et al. (29)	2015	50	42.39 ± 3.1	1 ml	Mixed	9	2	0	39	12
Wu et al. (30)	2015	49	NA	1 ml	Peritumoral	20	2	1	27	12
Mai et al. (31)	2015	43	NA	1 ml	Mixed	19	0	2	22	12
Wang et al. (32)	2015	41	NA	0.8 ml	Subareolar	16	3	1	21	12
Tu et al. (33)	2015	58	52.5 ± 13.1	0.5 ml	Subareolar	15	0	1	42	12
Guan et al. (34)	2015	87	NA	1 ml	Subareolar	31	0	2	54	12
Wu et al. (35)	2015	83	NA	NA	Subareolar	24	0	3	56	12
Lei et al. (36)	2014	56	NA	1 ml	Mixed	20	0	1	35	11
Ge et al. (37)	2013	88	NA	0.5 ml	Peritumoral	37	0	2	49	12
Gao et al. (38)	2013	34	NA	0.4 ml	Subareolar	14	0	2	19	11
Zhou et al. (39)	2012	74	NA	1 ml	Mixed	29	0	2	43	11
Chen et al. (40)	2012	44	NA	2 ml	Subareolar	22	0	2	20	12
Yang et al. (41)	2011	40	NA	2 ml	Subareolar	11	0	1	28	12
Li et al. (42)	2008	38	NA	2 ml	Subareolar	13	0	1	24	12

TP, true positive; FP, false positive; FN, false negative; TN, true negative; NA, not available; Mixed, the injection site is subareolar and peritumoral; QUADAS, quality assessment of diagnostic accuracy studies.

### Diagnostic Accuracy

**Figure 2:**
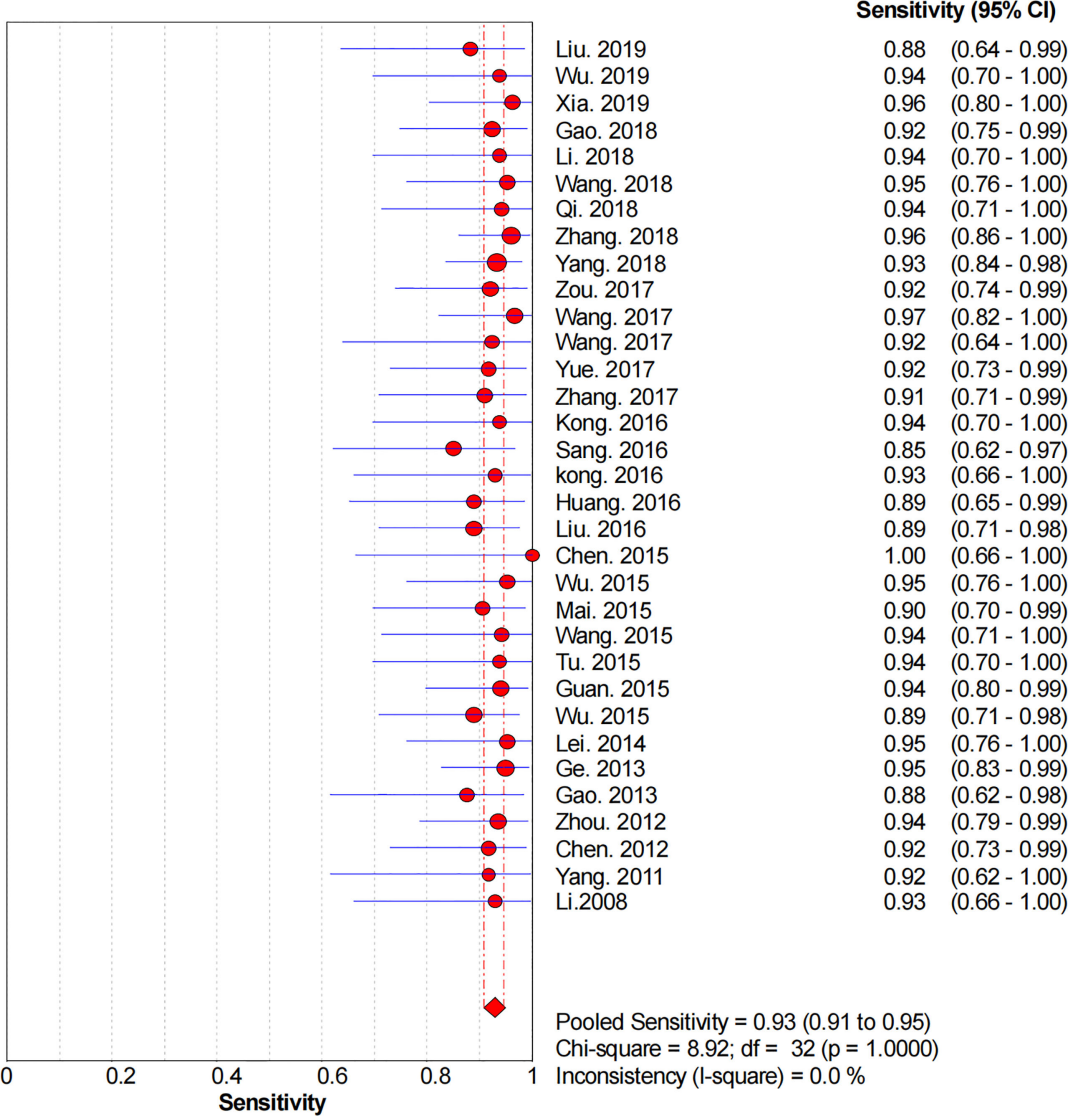
Forest plot of pooled sensitivity of the diagnosis value of CNSs in SLNB of breast cancer. 95% CI, 95% confidence interval.

**Figure 3:**
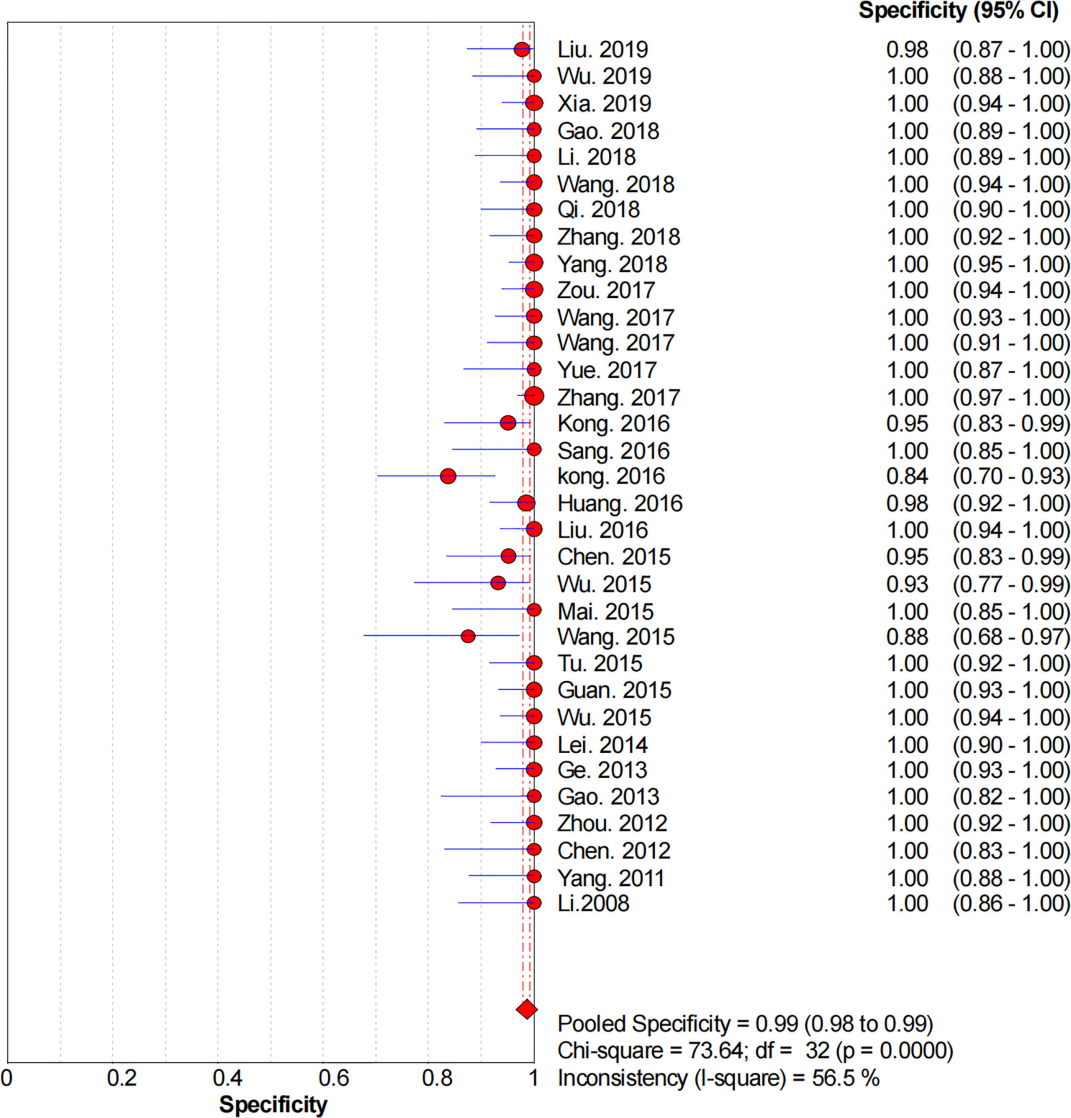
Forest plot of pooled specificity of the diagnosis value of CNSs in SLNB of breast cancer. 95% CI, 95% confidence interval.

**Figure 4:**
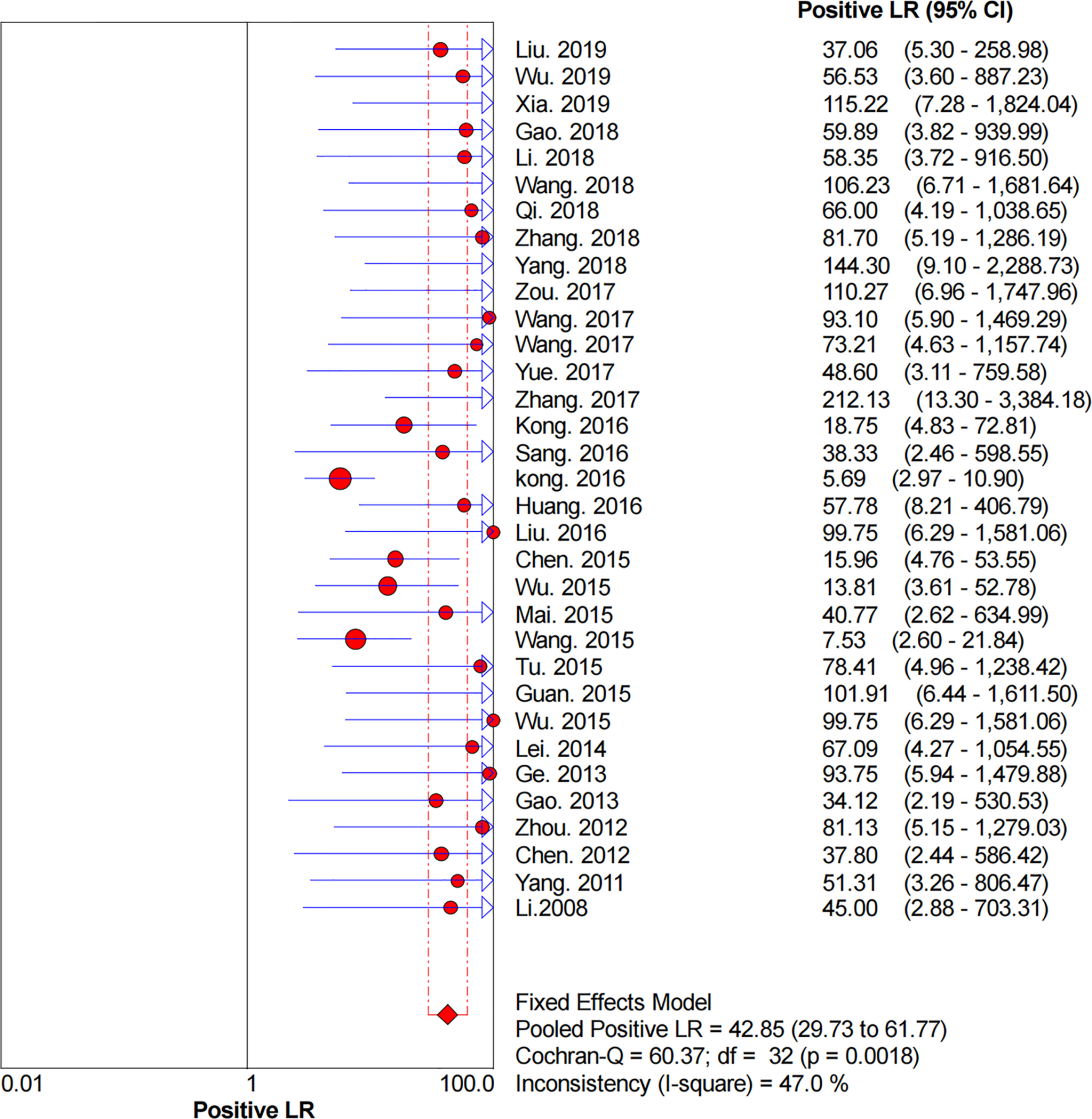
Forest plot of pooled PLR of the diagnosis value of CNSs in SLNB of breast cancer. 95% CI, 95% confidence interval; PLR, positive likelihood ratio.

**Figure 5:**
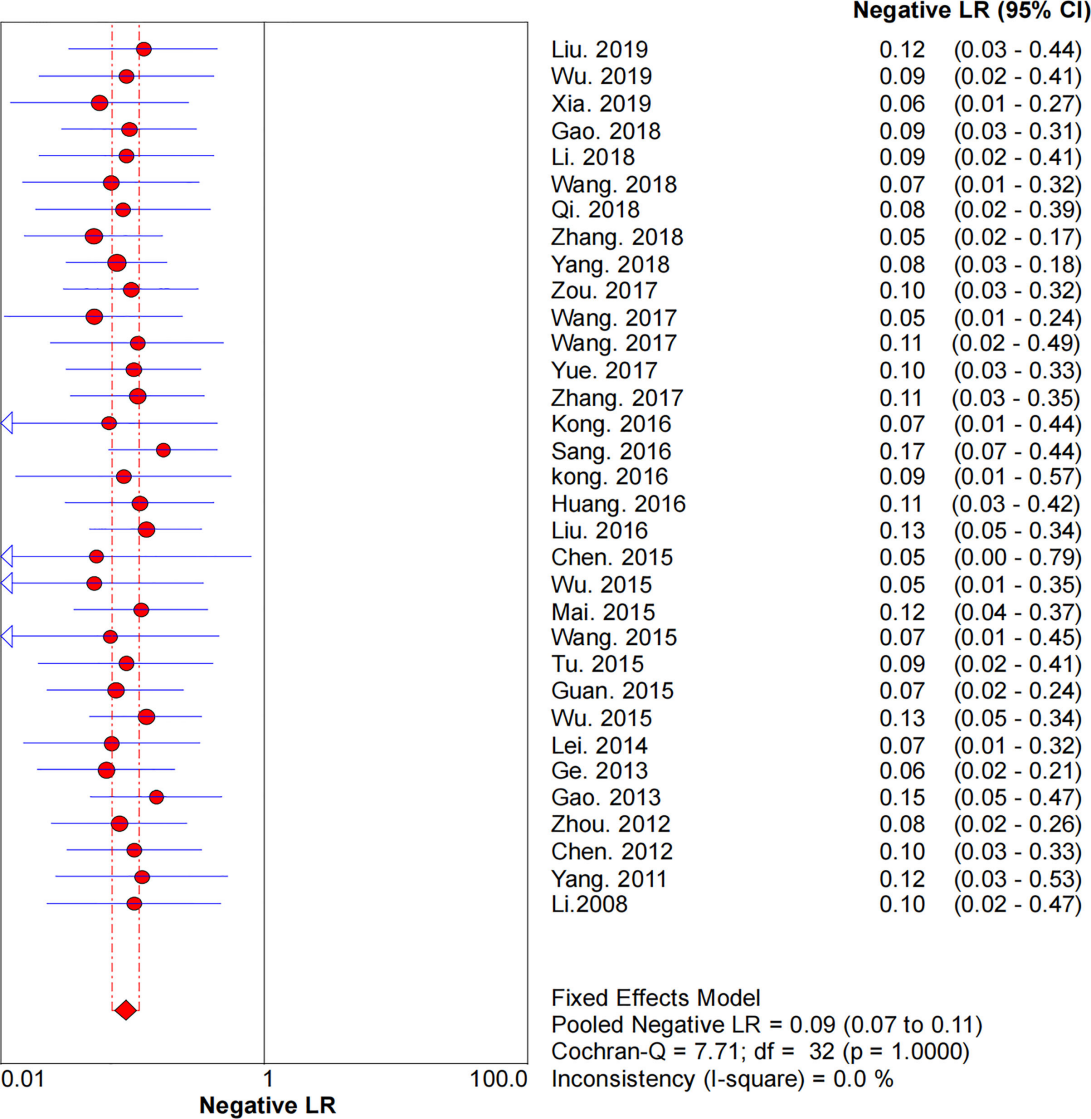
Forest plot of pooled NLR of the diagnosis value of CNSs in SLNB of breast cancer. 95% CI, 95% confidence interval; NLR, negative likelihood ratio.

**Figure 6:**
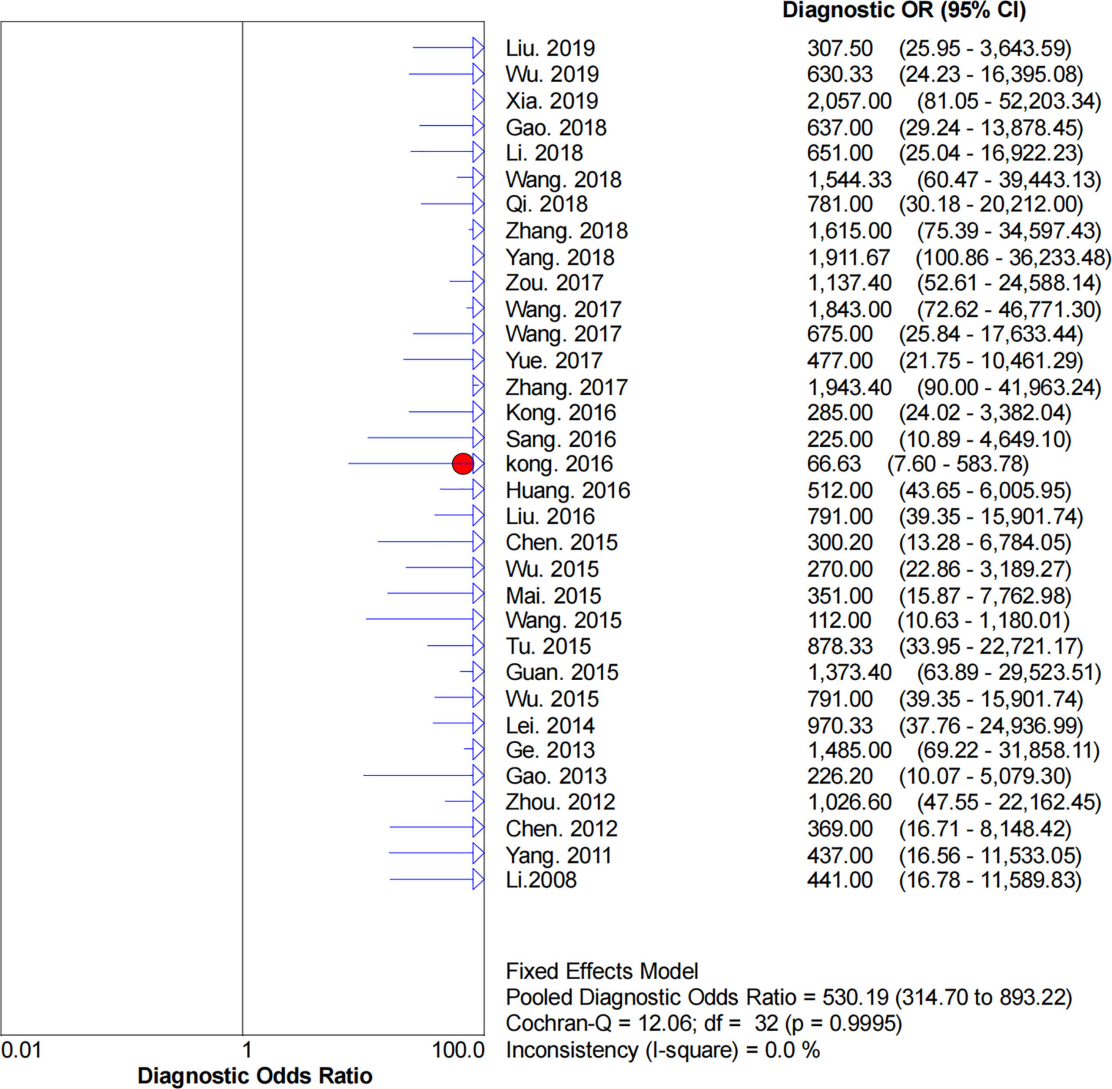
Forest plot of pooled DOR of the diagnosis value of CNSs in SLNB of breast cancer. 95% CI, 95% confidence interval. DOR, diagnostic odds ratio.

**Figure 7:**
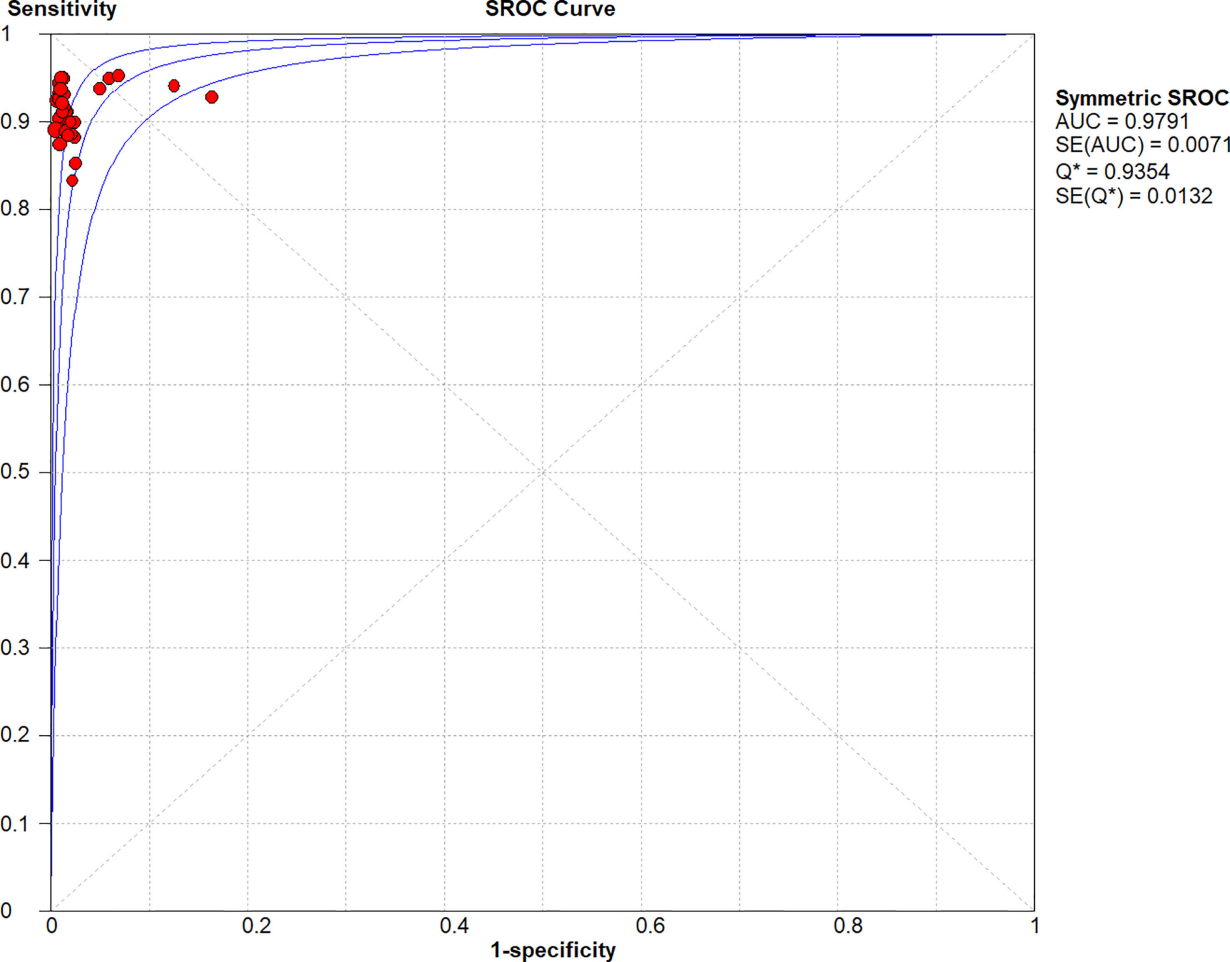
Symmetric SROC curve of the diagnosis value of CNSs in SLNB of breast cancer. SROC, summary receiver operating characteristic curve; AUC, the area under the receiver-operator characteristic curve.

**Figure 8:**
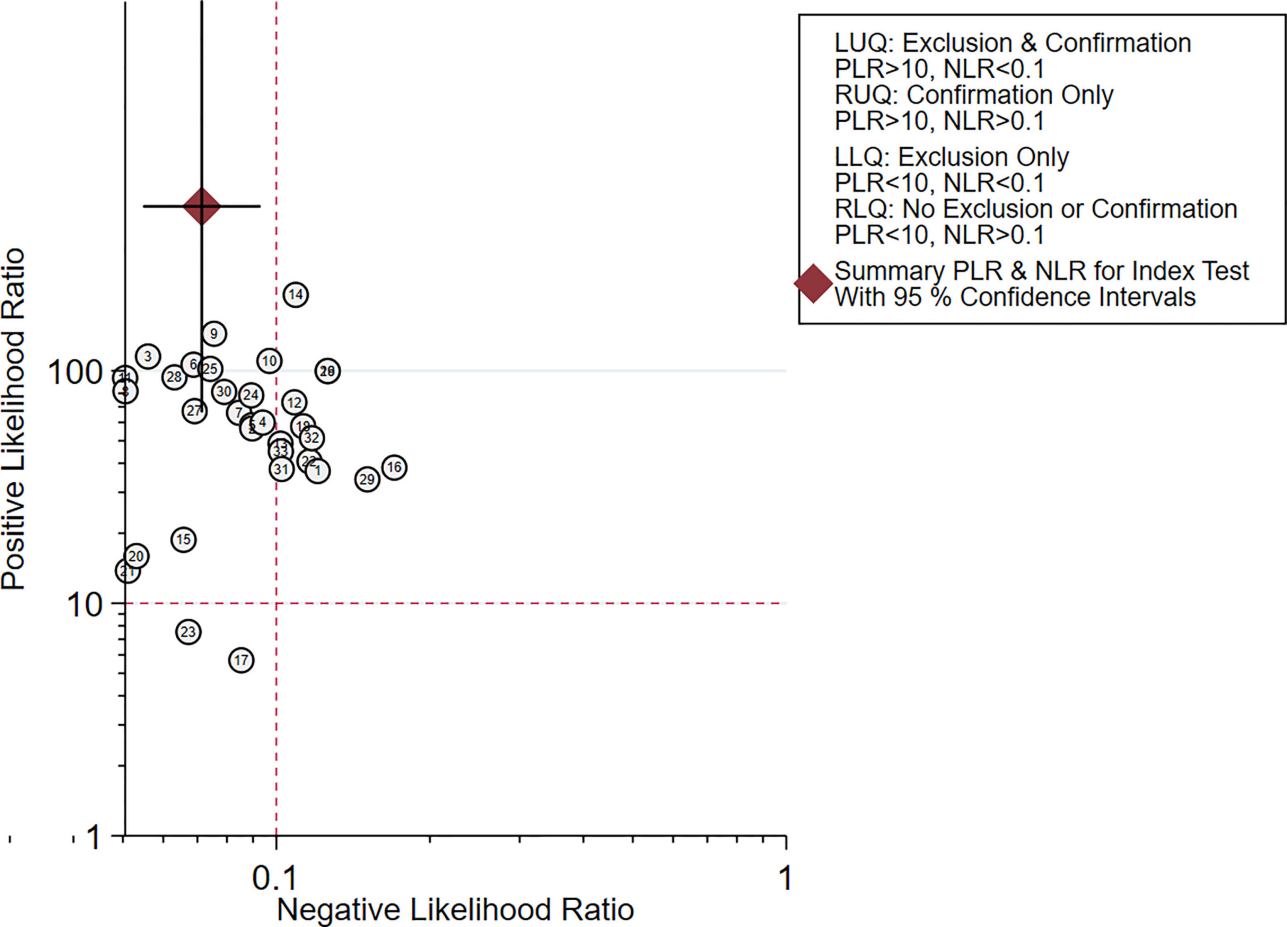
Scattergram of the PLR and NLR of the diagnosis value of CNSs in SLNB of breast cancer. PLR, positive likelihood ratio; NLR, negative likelihood ratio; LLQ, left lower quadrant; LUQ, left upper quadrant; RLQ, right lower quadrant; RUQ, right upper quadrant.

**Figures 2**–**6** demonstrate the forest plot of sensitivity, specificity, PLR, NLR, and DOR for CNS in SLNB. The overall pooled sensitivity and specificity of all studies were 0.93 (95% CI: 0.91–0.95, *I^2^* = 0.0%) and 0.99 (95% CI: 0.98–0.99, *I^2^* = 56.5%). The overall pooled PLR and NLR were 42.85 (95% CI: 29.73–61.77, *I^2^* = 47.0%) and 0.09 (95% CI: 0.07–0.11, *I^2^* = 0.0%), respectively. The pooled DOR was 530.19 (95% CI: 314.70–893.22, *I^2^* = 0.0%). The SROC curve demonstrated an AUC of 0.98, which indicated excellent diagnostic accuracy (**Figure 7**). Additionally, the left upper quadrant (LUQ) in the likelihood ratio scatter diagram was occupied by summary PLR and NLR, indicating that CNSs was useful in improving the diagnostic accuracy of SLNB in breast cancer (**Figure 8**).

There is controversy over the optimal dose and site of injection for the tracking agents. We compared the combined sensitivity and specificity of SLNB according to different CNSs doses (**Table 2**). For the studies that used a less than or equal to 1 ml injection of CNSs, the combined sensitivity was 0.93 (95% CI: 0.91–0.95, *I^2^* = 0.0%) and specificity was 0.98 (95% CI: 0.97–0.99, *I^2^* = 63.0%) (**Figure S1**). For the studies that used a 2 ml injection of CNSs, the combined sensitivity and specificity was 0.93 (95% CI: 0.87–0.97, *I^2^* = 0.0%) and 0.99 (95% CI: 0.97–1.00, *I^2^* = 9.3%) (**Figure S2**). The results suggested that the diagnostic value of CNSs was not dose-dependent over the range of doses tested.

**Table 2:** Subgroup analysis was performed based on Carbon Nanoparticle injection doses and site.

Subgroup	Sensitivity	Specificity	PLR	NLR	DOR
**Dose of CNSs (ml)**					
≤1 ml	0.93 (0.91–0.95)	0.98 (0.97–0.99)	39.09 (20.01–76.36)	0.08 (0.06–0.11)	510.16 (275.17–945.81)
2 ml	0.93 (0.87–0.97)	0.99 (0.97–1.00)	40.30 (16.24–100.03)	0.09 (0.05–0.16)	458.56 (145.49–1,445.35)
**Injection site**					
Subareolar	0.93 (0.89–0.95)	0.99 (0.98–1.00)	43.53 (24.56–77.14)	0.09 (0.07–0.13)	521.22 (244.10–1,112.97)
Peritumoral	0.94 (0.85–0.98)	0.98 (0.93–0.99)	31.93 (11.15–91.39)	0.08 (0.03–0.17)	476.71 (109.29–2,079.27)
Mixed	0.93 (0.90–0.96)	0.99 (0.97–0.99)	53.40 (18.17–156.95)	0.08 (0.06–0.12)	591.05 (252.32–1,384.52)

PLR, positive likelihood ratio; NLR, negative likelihood ratio; DOR, diagnostic odds ratio; CNSs, carbon nanoparticles suspensions; Mixed, the injection site is subareolar and peritumoral.

We further compared the effect of different injection sites, peritumoral or subareolar, on the SLNB (**Table 2**). The pooled sensitivity for studies that used subareolar injection was 0.93 (95% CI: 0.89–0.95, *I^2^* = 0.0%), while in studies using peritumoral and mixed injection, the pooled sensitivity was 0.94 (95% CI: 0.85–0.98, *I^2^* = 0.0%) and 0.93 (95% CI: 0.90–0.96, *I^2^* = 0.0%). The combined specificity for studies using subareolar, peritumoral and mixed injection was 0.99 (95% CI: 0.98–1.00, *I^2^* = 37.5%), 0.98 (95% CI: 0.93–0.99, *I^2^* = 50.6%) and 0.99 (95% CI: 0.97–0.99, *I^2^* = 71.2%), respectively. All groups were not significantly different from each other (**Figures S3–5**).

### Publication Bias and Sensitivity Analysis

**Figure 9:**
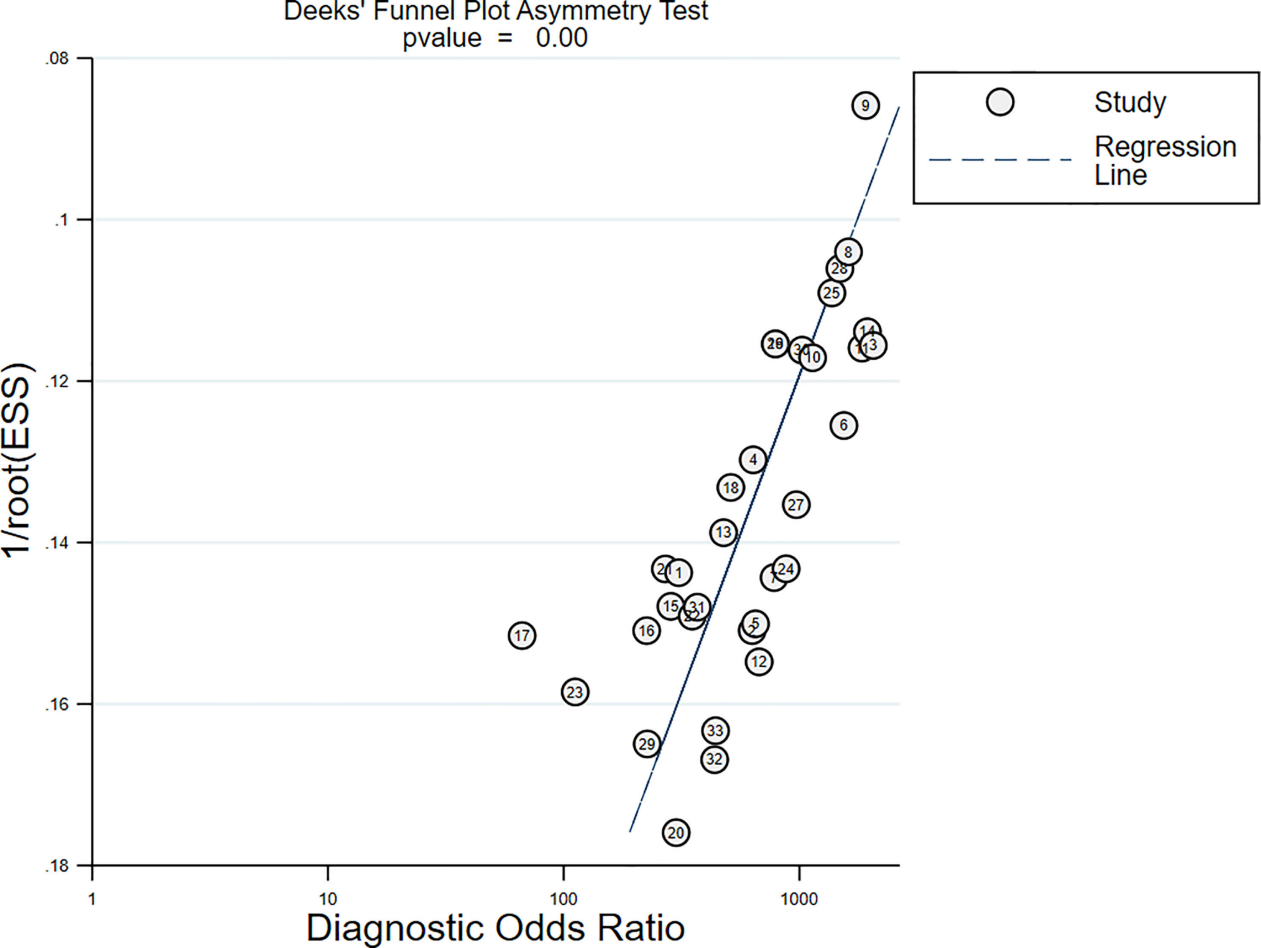
Deeks’ funnel plot for publication bias test.

All studies harbored significant publication bias, as indicated by the Deeks’ funnel plot [Slope (Bias) = −7.35, *P* = 0.00; **Figure 9**]. Nonetheless, the sensitivity analysis showed that the results were reliable and stable (**Table S1**).

## Discussion

SLNB was first reported in cutaneous melanoma by Morton et al. in 1992 (43). The SLNB concept was soon accepted for use in patients with breast cancer and led to better, less debilitating, axillary management (44). Both ALND and SLNB are not significantly different in terms of patient survival and tumor recurrence, thus further popularizing the widespread use of SLNB. SLNB carries the significant benefits of lower morbidity, especially with regards to arm lymphedema, paresthesia, and overall dysfunction (2–5). Currently, SLNB represents the standard surgical approach for axillary management in early breast cancer.

The mapping method is a decisive factor that determines the identification rate of SLN in breast cancer. RI technetium-99m was first used for SLNB mapping in 1993, followed by the use of blue dye (44, 45). The NSABP B-32 trial found that a combination of BD and radiocolloid resulted in a 97.1% detection rate for SNLB, compared with a 70.2% for BD and 89.4% for radiocolloid when used alone (46). Similar findings were noted in the ALMANAC study that demonstrated that a combination of isotope and BD had a 96.1% detection rate, but the use of either isotope or BD alone was 85.6% (47). Therefore, the method of combining BD and RI is currently regarded as the gold standard. Nevertheless, there are also disadvantages associated with this approach, namely, BD allergic reactions, the need for highly specialized nuclear medicine units, and the risk of radiation exposure to healthcare professionals and patients. New methods of lymphatic mapping that offer equal accuracy without the risks of allergies or irradiation are currently being trialed. A network meta-analysis showed that in contrast to using BD alone, superparamagnetic iron oxide nanoparticles or indocyanine green fluorescence alone are superior. The use of these novel agents alone is even comparable to the standard dual-modality technique. However, their use still mandates specialized equipment that may not be widely available (48).

CNSs is a new method that requires no specialized medical facilities for SLNB. This meta-analysis aimed to evaluate the diagnostic performance of CNSs for SLNB in breast cancer. The pooled sensitivity, specificity, and AUC of the SROC were 0.93, 0.99, and 0.98, respectively. The pooled DOR, a diagnostic performance index that takes into consideration specificity and sensitivity, in the current analysis was 530.19. Higher DOR values indicate a stronger discriminating power. The results suggest CNSs could be utilized to identify true positive patients with SLN metastases while also ruling out false negatives.

**Figure 10:**
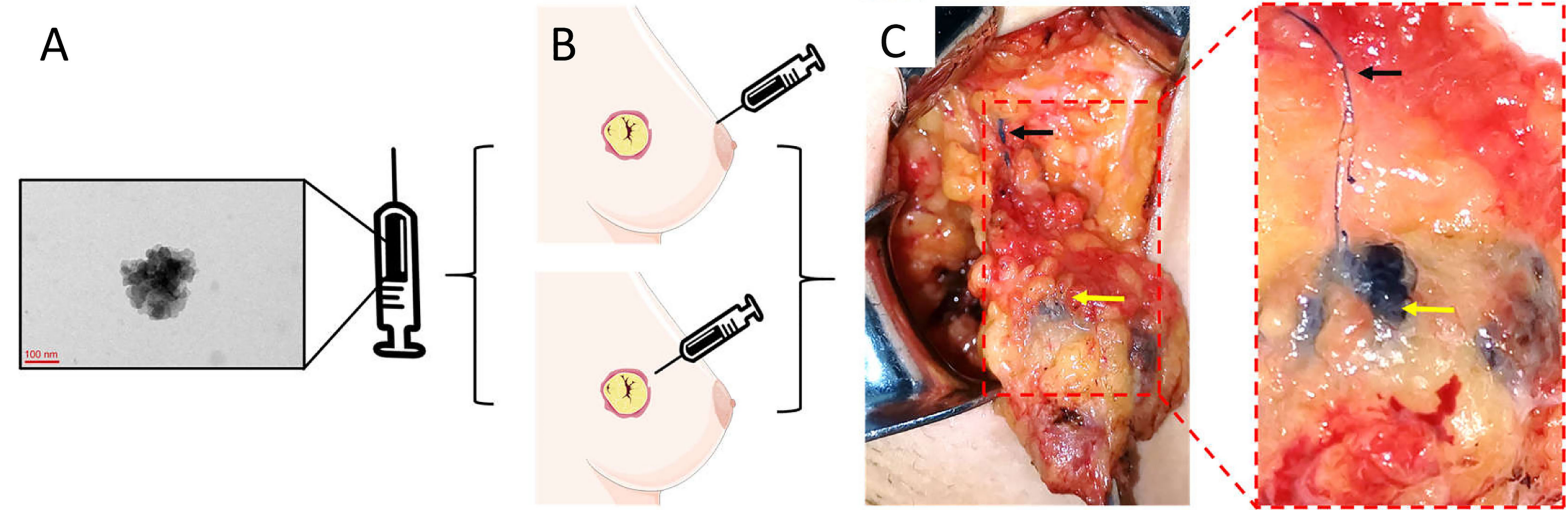
The specific operation steps of CNSs as lymphatic tracer in SLNB. **(A)** The morphology of CNSs; **(B)** Injection site of CNSs; **(C)** Color rendering of CNSs in SLNB (Black arrow: Lymphatic vessel; Yellow arrow: Lymph node). **(A)** is the image of CNSs under transmission electron microscopy. Republished with permission of SAGE Publications, Inc., from Liu X, Chang S, Jiang XL, Huang P, Yuan ZT. Identifying parathyroid glands with carbon nanoparticle suspension does not help protect parathyroid function in thyroid surgery: a prospective, randomized control clinical study. Surg Innov (2016) 23(4):381–9. doi: 10.1177/1553350615624787. ^©^ The Author(s) 2016; permission conveyed through Copyright Clearance Center, Inc (49).

The optimal dose and injection site of CNSs for SLNB is controversial. The most regularly used doses are 1 and 2 ml. In the 33 studies analyzed, the volume of CNSs varied from 0.2 to 2 ml (**Table 1**). Subgroup analysis highlighted that there was no difference in specificity or sensitivity between the studies that used ≤1 ml versus 2 ml injections of CNSs (**Table 2**), which indicated that 1 ml volume of CNSs is sufficient. In this meta-analysis, peritumoral CNSs injection for SLNB was used in 3 studies, subareolar CNSs injection was used in 15 studies, and 14 studies were used in both approaches. No significant difference in the sensitivity and specificity of SLNB was detected between studies using peritumoral and subareolar CNSs injection. Therefore, both peritumoral and subareolar are appropriate injection sites for SLNB with CNSs (**Figure 10**) (49).

In terms of adverse effects, none of the 2,171 included patients in this analysis developed a local inflammatory response, fat or skin necrosis, or an anaphylactic reaction. Nevertheless, the use of CNSs does have some limitations, with skin staining being the most frequently encountered side effect of CNSs (18, 35). This complication appears to be linked to the depth of injection based on our empirical observations. Therefore, a subcutaneous injection should be used instead of an intradermal injection. Another disadvantage of CNSs is that they cannot be seen through the skin and fatty tissue, therefore possessing lower visualization clarity compared to a fluorescent tracer (e.g., indocyanine green). Interestingly, a recent study suggests that CNSs have not only been employed as lymph node tracers but may also be useful as a carrier for antitumor therapy (50).

### Conclusions

This meta-analysis highlights the accuracy and feasibility of using CNSs for SLNB mapping in breast cancer patients. The CNSs mapping method would be especially helpful in institutions without access to fluorescence imaging systems or RI. CNSs may be incorporated in a wide range of clinical applications, namely, theranostics and in breast cancer therapy.

## Data Availability Statement

The original contributions presented in the study are included in the article/**Supplementary Material**. Further inquiries can be directed to the corresponding authors.

## Author Contributions

All authors read and approved the final manuscript prior to submission. YJ, JiL, BC, YB, CL, YL, and TL: data curation, software, writing—original draft. JuL and XC: supervision, writing—review and editing. All authors listed have made a substantial, direct, and intellectual contribution to the work and approved it for publication.

## Funding

This study was supported by the National Natural Science Foundation of China (No. 81860715) and the Doctor Foundation of Affiliated Hospital of Zunyi Medical University (No. 201712).

## Conflict of Interest

The authors declare that the research was conducted in the absence of any commercial or financial relationships that could be construed as a potential conflict of interest.

## Publisher’s Note

All claims expressed in this article are solely those of the authors and do not necessarily represent those of their affiliated organizations, or those of the publisher, the editors and the reviewers. Any product that may be evaluated in this article, or claim that may be made by its manufacturer, is not guaranteed or endorsed by the publisher.
