# Supplementary material for: Sentinel Lymph Node Biopsy Mapped With Carbon Nanoparticle Suspensions in Patients With Breast Cancer: A Systematic Review and Meta-Analysis
Source: Front Oncol. 2022 Mar 28;12:818812. doi: 10.3389/fonc.2022.818812 (PMC8995566; doi:10.3389/fonc.2022.818812)
Supplement: Supplementary file 1 [file DataSheet_1.docx]

**Supplementary Material**


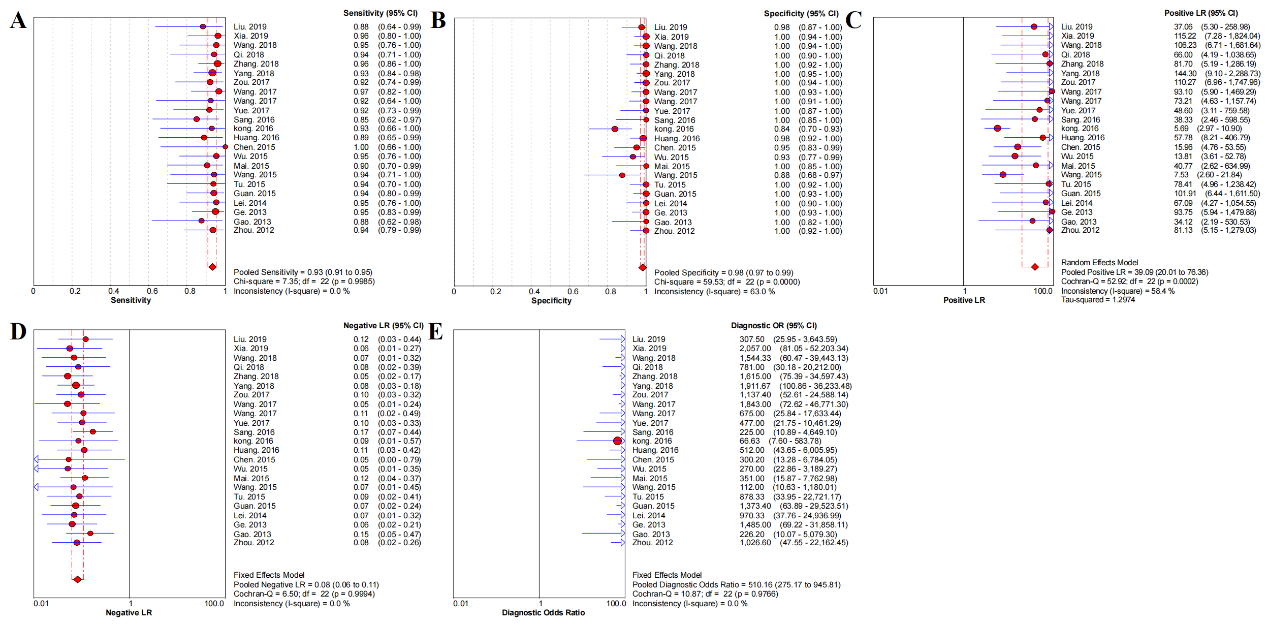


**FIGURE S1 |** Forest plot of sensitivity, specificity, PLR, NLR, DOR of SLNB according to used a less than or equal to 1 ml injection of CNSs. A: Sensitivity; B: Specificity; C: PLR; D: NLR; E: DOR. 95% CI, 95% confidence interval; PLR, positive likelihood ratio; NLR, negative likelihood ratio; DOR, diagnostic odds ratio.


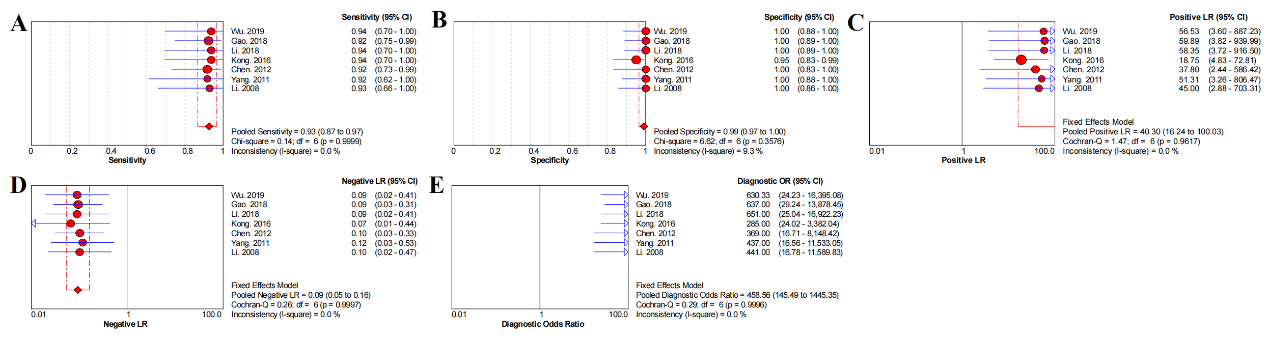


**FIGURE S2 |** Forest plot of sensitivity, specificity, PLR, NLR, DOR of SLNB according to used a 2 ml injection of CNSs. A: Sensitivity; B: Specificity; C: PLR; D: NLR; E: DOR. 95% CI, 95% confidence interval; PLR, positive likelihood ratio; NLR, negative likelihood ratio; DOR, diagnostic odds ratio.


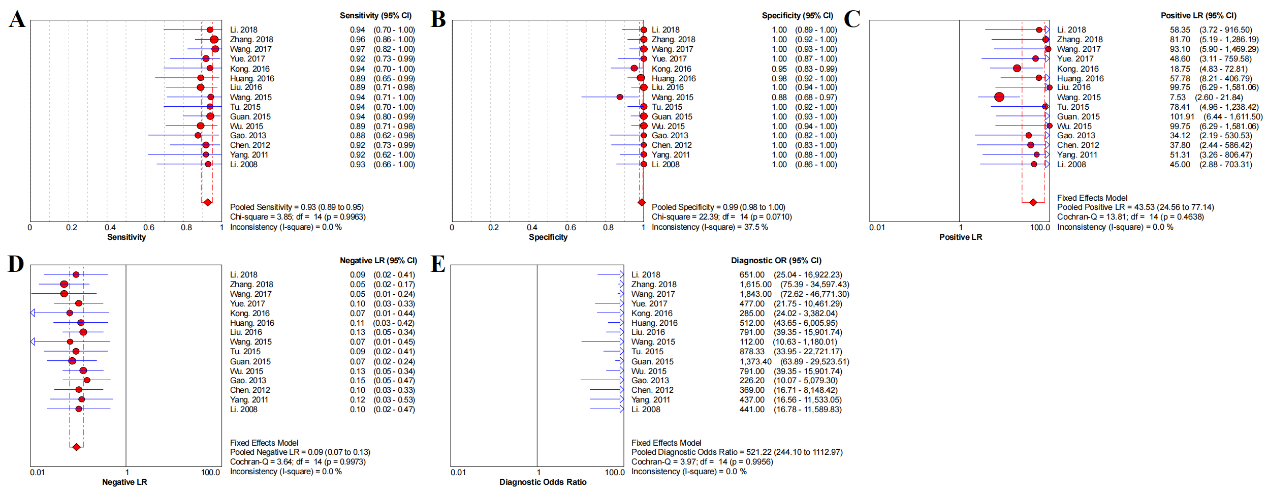


**FIGURE S3 |** Forest plot of sensitivity, specificity, PLR, NLR, DOR of SLNB according to used subareolar injection of CNSs. A: Sensitivity; B: Specificity; C: PLR; D: NLR; E: DOR. 95% CI, 95% confidence interval; PLR, positive likelihood ratio; NLR, negative likelihood ratio; DOR, diagnostic odds ratio.


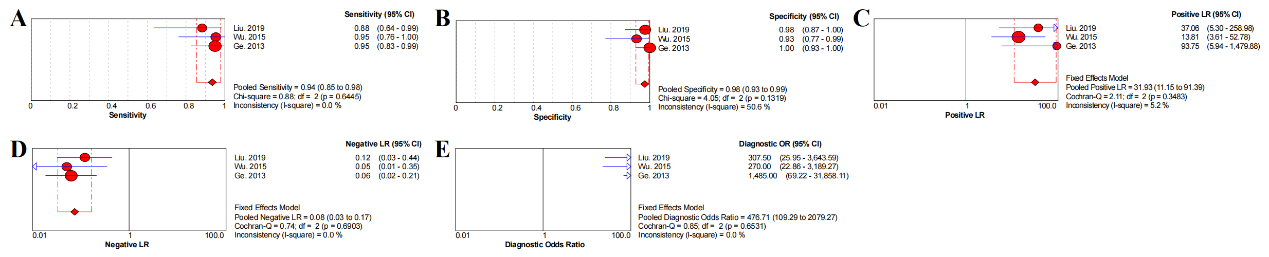


**FIGURE S4 |** Forest plot of sensitivity, specificity, PLR, NLR, DOR of SLNB according to used peritumoral injection of CNSs. A: Sensitivity; B: Specificity; C: PLR; D: NLR; E: DOR. 95% CI, 95% confidence interval; PLR, positive likelihood ratio; NLR, negative likelihood ratio; DOR, diagnostic odds ratio.


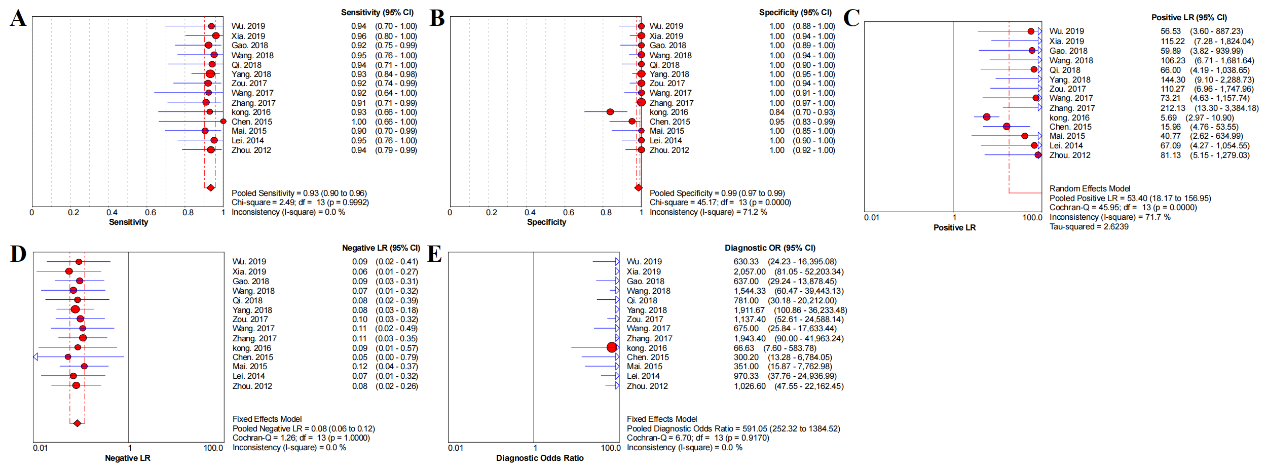


**FIGURE S5 |** Forest plot of sensitivity, specificity, PLR, NLR, DOR of SLNB according to used mixed injection of CNSs. A: Sensitivity; B: Specificity; C: PLR; D: NLR; E: DOR. 95% CI, 95% confidence interval; PLR, positive likelihood ratio; NLR, negative likelihood ratio; DOR, diagnostic odds ratio.

**TABLE S1** | Sensitivity analysis after each study was deleted.

| **Delete articles** | **DOR** | **Sensitivity** | **Specificity** |
| --- | --- | --- | --- |
| Liu et al. (10) | 539.82 | 0.93 | 0.99 |
| Wu et al. (11) | 528.23 | 0.93 | 0.99 |
| Xia et al. (12) | 514.11 | 0.93 | 0.99 |
| Gao et al. (13) | 527.39 | 0.93 | 0.99 |
| Li et al. (14) | 527.88 | 0.93 | 0.99 |
| Wang et al. (15) | 518.27 | 0.93 | 0.99 |
| Qi et al. (16) | 525.85 | 0.93 | 0.99 |
| Zhang et al. (17) | 512.06 | 0.93 | 0.99 |
| Yang et al. (18) | 502.08 | 0.93 | 0.99 |
| Zou et al. (19) | 519.33 | 0.93 | 0.99 |
| Wang et al. (20) | 514.77 | 0.93 | 0.99 |
| Wang et al. (21) | 527.73 | 0.93 | 0.99 |
| Yue et al. (22) | 531.80 | 0.93 | 0.99 |
| Zhang et al. (23) | 514.81 | 0.93 | 0.99 |
| Kong et al. (24) | 541.38 | 0.93 | 0.99 |
| Sang et al. (25) | 545.79 | 0.93 | 0.99 |
| Kong et al. (26) | 615.40 | 0.93 | 0.99 |
| Huang et al. (27) | 530.74 | 0.93 | 0.99 |
| Liu et al. (28) | 523.45 | 0.93 | 0.99 |
| Chen et al. (29) | 537.15 | 0.93 | 0.99 |
| Wu et al. (30) | 543.57 | 0.93 | 0.99 |
| Mai et al. (31) | 536.49 | 0.93 | 0.99 |
| Wang et al. (32) | 571.28 | 0.93 | 0.99 |
| Tu et al. (33) | 524.78 | 0.93 | 0.99 |
| Guan et al. (34) | 515.45 | 0.93 | 0.99 |
| Wu et al. (35) | 523.45 | 0.93 | 0.99 |
| Lei et al. (36) | 523.11 | 0.93 | 0.99 |
| Ge et al. (37) | 513.69 | 0.93 | 0.99 |
| Gao et al. (38) | 543.28 | 0.93 | 0.99 |
| Zhou et al. (39) | 520.00 | 0.93 | 0.99 |
| Chen et al. (40) | 535.73 | 0.93 | 0.99 |
| Yang et al. (41) | 532.27 | 0.93 | 0.99 |
| Li et al. (42) | 532.28 | 0.93 | 0.99 |

DOR, diagnostic odds ratio.
